# Nicotinic and opioid receptor regulation of striatal dopamine D2-receptor mediated transmission

**DOI:** 10.1038/srep37834

**Published:** 2016-11-25

**Authors:** Aphroditi A. Mamaligas, Yuan Cai, Christopher P. Ford

**Affiliations:** 1 Department of Neurosciences, Case Western Reserve University School of Medicine, 10900 Euclid Ave, Cleveland, OH, 44106-4970 USA; 2 Department of Physiology and Biophysics, Case Western Reserve University School of Medicine, 10900 Euclid Ave, Cleveland, OH, 44106-4970 USA

## Abstract

In addition to dopamine neuron firing, cholinergic interneurons (ChIs) regulate dopamine release in the striatum via presynaptic nicotinic receptors (nAChRs) on dopamine axon terminals. Synchronous activity of ChIs is necessary to evoke dopamine release through this pathway. The frequency-dependence of disynaptic nicotinic modulation has led to the hypothesis that nAChRs act as a high-pass filter in the dopaminergic microcircuit. Here, we used optogenetics to selectively stimulate either ChIs or dopamine terminals directly in the striatum. To measure the functional consequence of dopamine release, D2-receptor synaptic activity was assessed via virally overexpressed potassium channels (GIRK2) in medium spiny neurons (MSNs). We found that nicotinic-mediated dopamine release was blunted at higher frequencies because nAChRs exhibit prolonged desensitization after a single pulse of synchronous ChI activity. However, when dopamine neurons alone were stimulated, nAChRs had no effect at any frequency. We further assessed how opioid receptors modulate these two mechanisms of release. Bath application of the κ opioid receptor agonist U69593 decreased D2-receptor activation through both pathways, whereas the μ opioid receptor agonist DAMGO decreased D2-receptor activity only as a result of cholinergic-mediated dopamine release. Thus the release of dopamine can be independently modulated when driven by either dopamine neurons or cholinergic interneurons.

Striatal dopamine is a critical component of basal ganglia dependent movement and motivational behaviors^1^. Dopamine neurons exhibit a stereotyped burst-firing pattern in response to rewarding stimuli and their cues^1^,^2^,^3^ and provide synaptic input onto medium spiny neurons (MSNs), the main output neuron of the striatum^4^. In addition to dopamine neuron firing, striatal dopamine release can be driven by the activation of presynaptic nicotinic receptors (nAChRs) on dopamine terminals^5^,^6^,^7^,^8^,^9^. The major source of acetylcholine (ACh) in the striatum originates from local cholinergic interneurons (ChIs)^10^. Recent work has shown that optogenetic stimulation of ChIs is sufficient to drive the release of dopamine^5^,^11^, suggesting that synchronous activity of these striatal neurons evokes dopamine release independently of midbrain dopamine neuron impulse activity.

Studies using local electrical stimulation of the striatum have shown that inhibiting nicotinic receptors modulates the release of dopamine to a greater extent at low frequencies of stimulation^6^,^12^,^13^. This work suggests that presynaptic nAChRs may function to enhance the contrast of dopamine release during different patterns of dopamine neuron firing^12^,^13^. However, electrical stimulation simultaneously evokes dopamine release from both dopamine terminals and through ACh-mediated release. This pattern differs from the burst firing patterns of these two neurons, which occur out of phase. At rest, ChIs fire tonically at low frequencies (2–10 Hz)^4^,^14^. However, in response to both rewarding and aversive salient stimuli, ChIs exhibit a pause in tonic firing followed by a burst pattern that opposes reward-evoked dopamine neuron burst firing^15^,^16^. As a result, it still remains unclear how ChIs regulate dopamine release when dopamine terminals are stimulated independently.

To test the role of nicotinic receptors in frequency-dependent dopamine release onto D2-receptors in MSNs, we used optogenetics to separate the contribution of cholinergic and dopaminergic dopamine release while recording D2-receptor mediated inhibitory postsynaptic currents (D2-IPSCs). Overexpressed G-coupled inwardly rectifying potassium (GIRK) channels provided an electrophysiological sensor of D2-receptor activation in striatal MSNs^17^. Our results confirmed that nicotinic receptors potentiate dopamine release and subsequent D2-receptor activation in the striatum. However, due to rapid desensitization, nAChRs only facilitated dopamine release on the first stimulus of a synchronous burst. In addition, tonic firing of ChIs did not affect the release of dopamine when dopamine terminals were directly stimulated. Lastly, we found that due to the distinct expression patterns of opioid receptors in the striatum, mu opioid receptors (MORs) modulated only ChI-mediated dopamine transmission, while kappa opioid receptors (KORs) regulated transmission equally through both pathways.

## Experimental Procedures

### Stereotaxic injections

All animal procedures and protocols were approved by the Institutional Animal Care and Use Committee at Case Western Reserve University (CWRU IACUC, protocol # 2014-0012). All experiments were performed in accordance with the appropriate guidelines of the Institutional Animal Care and Use Committee at Case Western Reserve University.

Wild-type C57BL6, ChAT-internal ribosome entry site-Cre heterozygote (ChAT-Cre), or DAT-Cre transgenic heterozygote mice were injected at post-natal day 21. All animals were obtained from the Jackson Laboratory (Bar Harbor, ME). Sterotaxic surgeries were performed using a model 1900 stereotax (Kopf). Sterotaxic surgeries were performed under isoflurane anesthesia. Briefly, a small craniotomy was made using a 33-gauge drill bit above the desired coordinate. A small pulled glass pipette containing AAV was attached to a Nanoject II (Drummond) and was then inserted to the appropriate depth. Injections were performed at a rate of 90 nl/minute. The coordinates used for striatal injections were (relative to bregma): AP +1.15 mm, ML −1.85 mm, DV −3.325 mm. A volume of 300 nL of AAV9.hSyn.tdTomato.T2A.mGIRK2-1-A22A.WPRE.bGH was injected into one hemisphere of all mice. To optogenetically activate ChIs, 300 nL of AAV5.EF1a.DIO.hChR2(H134R)-EYFP.WPRE.hGH was combined with the 300 nL of AVV.GIRK2 and injected at the same time in ChAT-Cre mice. To optogenetically activate dopamine terminals, 500 nL of AAV5.EF1a.DIO.hChR2(H134R)-EYFP.WPRE.hGH was separately injected into one side of the midbrain of DAT-Cre mice. Midbrain coordinates for the SNc/VTA (relative to bregma) were: AP −2.3 mm, ML −0.55 mm, DV −4.7 mm. All AAVs were obtained from the University of Pennsylvania Viral Core. Animals were allowed to recover for ~2–4 weeks to allow for viral expression.

### Slice preparation

Coronal slices (240 μM) containing the striatum were made in ice-cold cutting solution containing (in mM): 75 NaCl, 2.5 KCl, 6 MgCl_2_, 0.1 CaCl_2_, 1.2 NaH_2_PO_4_, 25 NaHCO_3_, 2.5 D-glucose, and 50 sucrose; bubbled with 95% O_2_ and 5% CO_2_. Slices were incubated at 35 °C for at least 45 minutes in ACSF containing (in mM): 126 NaCl, 2.5 KCl, 1.2 MgCl_2_, 2.4 CaCl_2_, 1.2 NaH_2_PO_4_, 21.4 NaHCO_3_, and 11.1 D-glucose; and 10 μM MK-801 bubbled with 95% O_2_ and 5% CO_2_. Slices were transferred to a recording chamber and perfused with warm ACSF (34 ± 2 °C) at 2 mL/minute. Perfusion solution contained picrotoxin (100 μM), DNQX (10 μM), CGP 55845 (300 nM), SCH 23390 hydrochloride (1 μM), and scopolamine (500 nM) to block GABA_A_, AMPA, GABA_B_, dopamine D1 and muscarinic receptors respectively. MSNs were visualized using a BXWI51 scope (Olympus) with custom-built gradient contrast optics using a near-IR LED (Thor Labs).

### Electrophysiology

Whole-cell recordings were performed using either Axopatch 200 A or Axopatch 200B amplifiers (Molecular Devices). Membrane potentials were not corrected for liquid junction potentials. Patch pipettes (1.5–2 MΩ) were made from borosciliate glass capillary tubes (World Precision Instruments). Patch pipettes for MSNs contained (in mM): 115 K-methylsulfate, 20 NaCl, 1.5 MgCl_2_, 10 HEPES(K), 10 BAPTA-tetrapotassium. Patch pipettes for ChIs contained (in mM): 135 D-gluconate(K), 10 HEPES(K), 0.1 CaCl_2_, 2 MgCl_2_, 0.1 EGTA. All internal patch solution contained: 1 mg/mL ATP, 0.1 mg/mL GTP, and 1.5 mg/mL phosphocreatine (pH 7.35, 275 mOsm). Membrane potential was held at −60 mV during voltage clamp recordings. Cells were discarded if their series resistance exceeded 15 MΩ, as series resistance was not compensated. ChIs were identified by the presence of an h-current when stepped to −90 mV. Data was acquired with Axograph X (Axograph Scientific) at 5 kHz. Voltage clamp recordings were filtered to 2 kHz. Electrical stimulation (0.7 ms) was applied with a monopolar extracellular stimulating electrode filled with ACSF (World Precision Instruments). Optogenetic stimulation was evoked with 2 ms widefield illumination (470 nm) using a custom made LED (470 nm Rebel LED Star Saber, Luxeon Star). All drugs were applied via bath application unless otherwise specified. All recordings were made in the dorsal striatum.

### Materials

DNQX, picrotoxin, MK-801, and naloxone were obtained from Ascent Scientific. SCH 23390, CGP 55845, scopolamine hydrobromide, ambenonium, dihydro-b-erythroidine hydrobromide (DHβE), mecamylamine, sulpiride, and DAMGO were obtained from Tocris Bioscience. K-methylsulfate was from Acros Organic. BAPTA was from Invitrogen. All other chemicals were from Sigma-Aldrich.

### Statistics and analysis

Data are shown as mean ± SEM. Statistical significance was assessed using either Wilcoxon matched-pairs signed rank test, Mann-Whitney test, or one-way ANOVA where appropriate (InStat 3.0, Graphpad).

## Results

### Activation of D2 receptors in MSNs

To examine D2-receptor activation in response to nicotinic receptor activity, we overexpressed G-protein coupled inwardly rectifying K^+^ (GIRK2) channels in striatal MSNs^17^. Injection of an adeno-associated virus (AAV) encoding GIRK2 and a soluble td-Tomato fluorophore resulted in expression of both proteins in MSNs^17^,^18^. After allowing three weeks for expression, coronal striatal slices were obtained from AAV-injected mice. Viral expression was observed in both direct and indirect pathway MSNs^17^,^18^, but was restricted from ChIs likely as a result of poor efficiency of transfection of ChIs using a synapsin promoter^18^. It was not examined whether other GABAergic interneurons expressed GIRK following AAV injection. All experiments in the present study were performed in the presence of antagonists for GABA_A_, GABA_B_ AMPA, NMDA, dopamine D1, and muscarinic receptors. Td-Tomato^+^ MSNs were voltage-clamped at a holding potential of −60 mV. As about half of striatal MSNs belong to the D2-receptor expressing indirect pathway^19^, D2-receptor mediated GIRK2 currents were observed in roughly half of GIRK2^+^ MSNs^17^.

**Figure 1:**
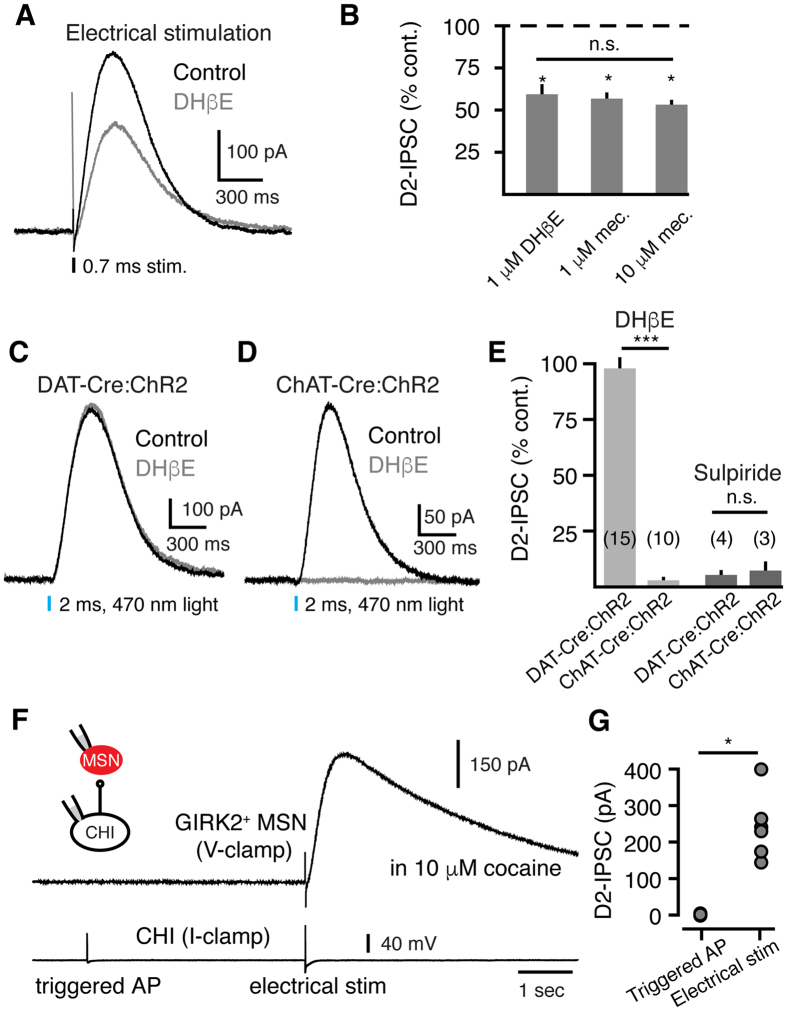
Nicotinic receptor modulation of D2-receptor activation. (**A**) Antagonism of α4β2 nicotinic receptors with DHβE (1 μM) attenuates electrically evoked D2-IPSCs. A single electrical stimulation (0.7 ms) was used to evoke the release of dopamine. (**B**) Quantification of electrically evoked D2-IPSC inhibition by the nAChR antagonists DHβE (1 μM) and mecamylamine (1 μM and 10 μM) (*p < 0.05, Wilcoxon matched-pairs signed rank and one-way ANOVA). (**C**) Optogenetic activation of dopamine terminals expressing ChR2 evokes D2-IPSCs in GIRK2^+^ MSNs (DAT-Cre:ChR2, 2 ms flash, 470 nm light). D2-IPSCs evoked by photostimulation of dopamine terminals were unaffected by DHβE (1 μM, gray trace). (**D**) Synchronous optogenetic activation of striatal ChIs expressing ChR2 is sufficient to evoke D2-IPSCs in GIRK2^+^ MSNs (ChAT-Cre:ChR2, 2 ms flash, 470 nm light). D2-IPSCs evoked by photostimulation of ChIs were abolished by DHβE (1 μM, gray trace). (**E**) Summary data quantifying the effect of DHβE (1 μM) and the D2-receptor antagonist sulpiride (400 nM) on D2-IPSCs optogenetically evoked by dopamine terminal stimulation (DAT-Cre:ChR2) or ChI stimulation (ChAT-Cre:ChR2) (**p < 0.01, Mann-Whitney). (**F**) In paired recordings between ChIs and GIRK2^+^ MSNs done in the presence of 10 μM cocaine, paired D2-IPSCs were not resolvable. Electrical stimulation of the surrounding region evoked a robust D2-IPSC. ChIs were recorded in current clamp, while MSNs were recorded in voltage clamp. (**G**) Summary data illustrating the amplitudes of action potential evoked D2-IPSCs and electrically evoked D2-IPSCs in 10 μM cocaine (*p < 0.05, Wilcoxon matched-pairs signed rank).

Electrical stimulation within the striatum evoked a D2-receptor mediated inhibitory post-synaptic current (D2-IPSC) in indirect pathway GIRK2^+^ MSNs that was attenuated by the α4β2 nicotinic receptor subtype antagonist DHβE (1 μM; 40.6 ± 5% inhibition, n = 5, p < 0.05, W = 28, Wilcoxon) (Fig. 1A and B). Saturating concentrations of the broad spectrum nicotinic antagonist mecamylamine similarly inhibited D2-IPSCs by roughly half (1 μM: 42.5 ± 2% inhibition, n = 5, W = −15; 10 μM: 46.8 ± 2% inhibition, n = 6, W = −21; p < 0.05 for both concentrations, Wilcoxon) (Fig. 1B). As α7 subunit-containing nicotinic receptors are not abundantly present in the dorsal striatum^20^, mecamylamine and DHβE produced similar inhibition of D2-IPSCs (p > 0.05, One-way ANOVA). Bath application of ambenonium, an acetylcholinesterase inhibitor, also caused a decrease in the amplitude of D2-IPSCs (100 nM; 32.2 ± 2% inhibition, n = 8, p < 0.01, W = −36, Wilcoxon). Electrochemical studies have found that nicotinic receptor antagonism evokes a substantial decrease in dopamine release^6^,^12^,^13^. Similarly, the observed ~40% decrease in electrically evoked D2-IPSCs shows that saturating concentrations of nicotinic receptor antagonists are insufficient to completely eliminate D2-receptor activation. Thus, electrical stimulation both directly stimulates midbrain terminals and also drives the di-synaptic release of dopamine through the activation of presynaptic nAChRs on dopamine terminals at D2-receptor synapses.

To separate cholinergic-mediated dopamine release from direct dopamine terminal driven release, we employed an optogenetic approach. We injected a double-floxed virus encoding the light activated cation channel channelrhodopsin-2 (ChR2, AAV.DIO.ChR2.eYFP) into either the substantia nigra (SNc) of DAT-Cre mice or into the striatum of ChAT-IRES-Cre mice. Photostimulation of either dopamine terminals (DAT-Cre:ChR2) or ChIs (ChAT-Cre:ChR2) with a single flash of blue light (470 nm, 2 ms) was sufficient to evoke D2-IPSCs in indirect pathway GIRK2^+^ MSNs (Fig. 1C and D). In both cases, IPSCs were eliminated by the D2-receptor antagonist sulpiride (400 nM; n = 4; p < 0.05, U = 4, Mann-Whitney) (Fig. 1E) and tetrodotoxin (200 nM; ChAT-Cre:ChR2: 16.1 ± 3 pA, n = 4; DAT-Cre:ChR2: 17.4 ± 3 pA, n = 5). DHβE (1 μM) eliminated ChAT-Cre:ChR2 evoked D2-IPSCs (n = 10, p < 0.01 relative to control, W = −55, Wilcoxon) (Fig. 1D and E) but did not alter DAT-Cre:ChR2 evoked D2-IPSCs (n = 15, p > 0.05, W = −32, Wilcoxon; p < 0.0001 versus the inhibition of ChAT-Cre:ChR2 evoked IPSCs, U = 0, Mann-Whitney) (Fig. 1C and E). Thus, while synchronous activity of ChIs can drive the release of dopamine through nicotinic receptors^5^,^11^, the background tonic firing of ChIs did not modulate the release of dopamine when SNc terminals were directly activated.

We next examined whether a single action potential in a ChI was sufficient to evoke D2-receptor activation. To test this, we made paired recordings of ChIs (current clamp) and GIRK2^+^-expressing MSNs (voltage clamp). ChIs were hyperpolarized to just below threshold during these recordings to prevent tonic firing. To increase the resolvable amplitude of paired IPSCs, dopamine transporters were inhibited with the dopamine transport blocker cocaine (10 μM). While electrical stimulation evoked robust D2-IPSCs, single action potentials in ChIs failed to elicit D2-IPSCs (Fig. 1F and G). Single action potentials also did not evoke an IPSC when recorded in the presence of ambenonium (100 nM), which inhibits acetylcholinesterase thereby increasing the concentration of striatal ACh (4.7 ± 2 pA, n = 5). This confirms that multiple ChIs activated together are required to engage nicotinic receptor mediated dopamine release^11^.

### Frequency dependence of cholinergic-induced dopamine release

**Figure 2:**
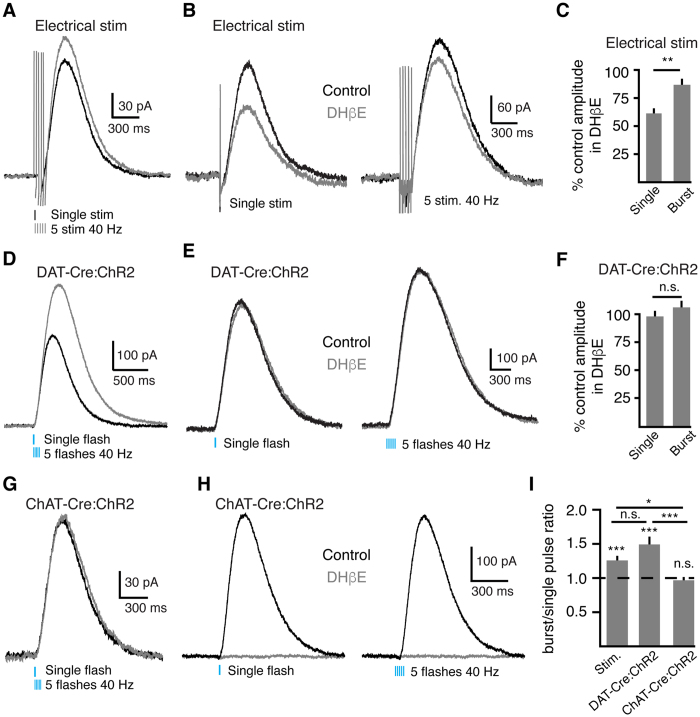
Frequency dependence of D2-IPSCs evoked by dopamine terminal or ChI stimulation. (**A**) Representative traces of D2-IPSCs evoked by electrical stimuli. Illustrated are IPSCs evoked by a single stimulus (black) and bursts (5 at 40 Hz, gray). (**B**) D2-IPSCs evoked by a single electrical stimulus are inhibited to a greater extent by DHβE (1 μM) than D2-IPSCs evoked with bursts of stimuli (5 at 40 Hz). (**C**) Quantification of DHβE inhibition of D2-IPSCs evoked by single or bursts of electrical stimuli (**p < 0.01, Mann-Whitney). (**D**) Representative traces of D2-IPSCs evoked by photostimulation of dopamine terminals. Illustrated are IPSCs evoked by a single stimulus (black) and bursts (5 at 40 Hz, gray). (**E**) D2-IPSCs evoked by photostimulation of dopamine terminals (DAT-Cre:ChR2) show little inhibition by DHβE (1 μM). (**F**) Quantification of the lack of effect of DHβE (1 μM) on D2-IPSCs evoked by photostimulation of dopamine terminals. (Mann-Whitney). (**G**) Representative traces of D2-IPSCs evoked by photostimulation of ChIs. Illustrated are IPSCs evoked by a single stimulus (black) and bursts (5 at 40 Hz, gray). (**H**) D2-IPSCs evoked by photostimulation of ChIs (ChAT-Cre:ChR2) are abolished in the presence of DHβE (1 μM). (**I**) Quantification of amplitude of D2-IPSCs evoked by bursts of stimulation normalized to the amplitude of single stimuli (*p < 0.05, ***p < 0.001, Wilcoxon matched-pairs signed rank within group and Mann-Whitney between groups).

Past work has demonstrated that nicotinic receptors provide stronger modulation of dopamine release during low frequency electrical stimulation^12^. To examine this through the activation of D2-receptors, we compared the effect of a single electrical stimulus to bursts of stimuli (5 at 40 Hz). Bursts evoked larger amplitude D2-IPSCs than single stimuli (burst 125.8 ± 5% of single stimulation amplitude, n = 14, p < 0.001, W = 91, Wilcoxon) (Fig. 2A and I)^21^. In addition, DHβE (1 μM) inhibited electrically evoked D2-IPSCs less when driven by bursts than single stimuli (single: 39.2 ± 4% inhibition, n = 4; burst: 13 ± 4% inhibition, n = 6; p < 0.01, Mann-Whitney) (Fig. 2B and C). This indicates that ChI-mediated dopamine transmission contributed less during high frequency stimulation and is consistent with the high-pass filter model describing nicotinic modulation of dopamine release that has been seen before with electrical stimulation^12^.

*In vivo*, ChIs and dopamine neurons fire in opposing patterns during reward-learning tasks^15^,^16^. Phasic firing of dopamine neurons drives pauses in the tonic activity of ChIs through the activation of D2-receptors^22^,^23^,^24^,^25^. As ChIs and dopamine neurons do not fire bursts at the same time, we next examined the frequency dependence of dopamine release driven by optogenetic activation of dopamine terminals or ChIs individually. In DAT-Cre:ChR2 expressing mice, bursts of photostimulation of dopamine terminals evoked D2-IPSCs that were larger in amplitude relative to single flashes (DAT-Cre:ChR2: 149.2 ± 10% of single flash amplitude, n = 15, p < 0.0001, W = 120, Wilcoxon) (Fig. 2D and I). DHβE (1 μM) did not alter the amplitude of DAT-Cre:ChR2-evoked D2-IPSCs (p > 0.05, n = 13–15, U = 72, Mann-Whitney) (Fig. 2E and F). Thus, tonic nicotinic receptor activity is insufficient to modulate dopamine release at D2-receptor synapses. In contrast, bursts of photostimulation of ChIs in ChAT-Cre:ChR2-expressing slices did not potentiate D2-IPSCs (ChAT-Cre:ChR2: 96.8 ± 3% of single flash amplitude, n = 11, p > 0.05, W = −16, Wilcoxon) (Fig. 2G and I). In all cases, DHβE (1 μM) eliminated ChI-driven D2-IPSCs (bursts: 4.0 ± 1% of baseline, p < 0.05, n = 5, W = −15) (Fig. 2H). Thus, cholinergic-mediated dopamine release does not facilitate with bursts. As a result, nicotinic receptors only facilitate dopamine release during the first pulse of synchronous ChI burst activity.

**Figure 3:**
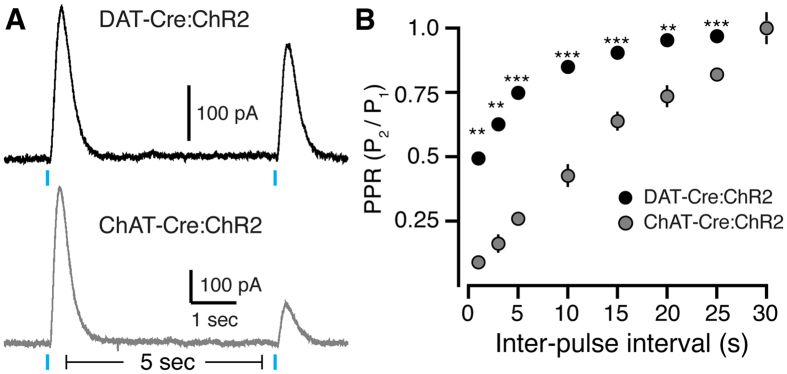
Paired pulse ratio shows slow recovery of D2-IPSCs evoked by photostimulation of ChIs. (**A**) Representative traces illustrating optogenetic paired pulse stimulation of dopamine terminals (DAT-Cre:ChR2, top) and ChIs (ChAT-Cre:ChR2, bottom). At a 5 second inter-pulse interval, D2-IPSCs evoked by ChI photostimulation have a lower paired pulse ratio relative to IPSCs evoked by photostimulation of dopamine terminals. (**B**) Quantification of paired pulse ratio (P_2_/P_1_) for both DAT-Cre:ChR2 evoked D2-IPSCs and ChAT-Cre:ChR2 evoked D2-IPSCs at inter-pulse intervals of 1, 3, 5, 10, 15, 20, 25, and 30 seconds (**p < 0.01, ***p < 0.001, Mann-Whitney).

To determine the kinetics of nAChR desensitization, we next examined the time course of recovery of D2-IPSCs. Paired pulse experiments revealed that ChAT-Cre:ChR2 evoked IPSCs took significantly longer to recover than DAT-Cre:ChR2 evoked IPSCs (Fig. 3). As D2-IPSCs evoked by direct dopamine terminal stimulation recovered faster than D2-IPSCs evoked by ChI-stimulation, (Fig. 3B), the long recovery of ChAT-Cre:ChR2 evoked D2-IPSCs was likely not due to intrinsic properties of dopamine release. We found that ChAT-Cre:ChR2 IPSCs evoked at a 1 second interpulse interval were almost completely abolished, while DAT-Cre:ChR2 IPSCs only decreased by half their amplitude (1 sec PPR: DAT 0.49 ± 0.02, ChAT 0.09 ± 0.01, n = 6 for both groups, p < 0.01, U = 0, Mann-Whitney). This difference in PPR suggests that nicotinic receptors on SNc terminals likely exhibit a slow recovery from desensitization in response to synchronous ACh release^26^. Together, these data indicate that nicotinic receptors on dopamine terminals positively modulate D2-receptor transmission during synchronous ChI firing yet desensitize at high frequencies.

### Opioid modulation of striatal D2-IPSCs in MSNs

**Figure 4:**
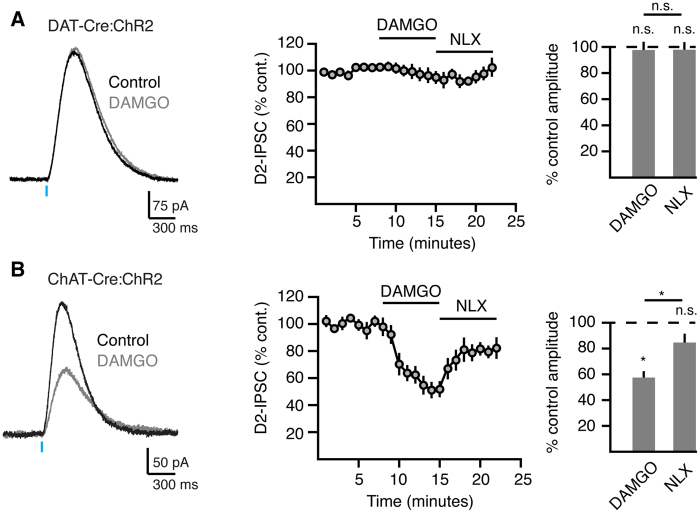
Mu opioid receptors selectively regulate ChI-mediated dopamine release. (**A**) The effect of the MOR agonist DAMGO (1 μM) on D2-IPSCs evoked by photostimulation of dopamine terminals (DAT-Cre:ChR2). Left: representative trace, middle: normalized quantification of D2-IPSC amplitude over the time, right: bar graph quantifying the amplitude of D2-IPSCs following bath application of DAMGO (1 μM), and naloxone (1 μM) (Wilcoxon matched-pairs signed rank). (**B**) The effect of the MOR agonist DAMGO (1 μM) on D2-IPSCs evoked by photostimulation of ChIs (ChAT-Cre:ChR2). Left: representative trace, middle: normalized quantification of D2-IPSC amplitude over the time, right: bar graph quantifying the amplitude of D2-IPSCs following bath application of DAMGO (1 μM), and naloxone (1 μM) (Wilcoxon matched-pairs signed rank).

**Figure 5:**
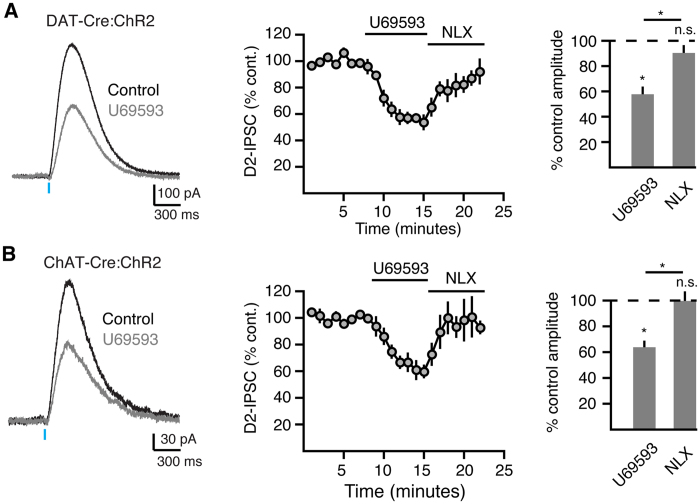
Kappa opioid receptors inhibit D2-IPSCs evoked by both dopamine terminal and ChI stimulation. (**A**) The effect of the KOR agonist U69593 (300 nM) on D2-IPSCs evoked by photostimulation of dopamine terminals (DAT-Cre:ChR2). Left: representative trace, middle: normalized quantification of D2-IPSC amplitude over the time, right: bar graph quantifying the amplitude of D2-IPSCs following bath application of U69593 (300 nM), and naloxone (1 μM) (*p < 0.05, Wilcoxon matched-pairs signed rank). (**B**) The effect of the KOR agonist U69593 (300 nM) on D2-IPSCs evoked by photostimulation of ChIs (ChaT-Cre:ChR2). Left: representative trace, middle: normalized quantification of D2-IPSC amplitude over the time, right: bar graph quantifying the amplitude of D2-IPSCs following bath application of U69593 (300 nM), and naloxone (1 μM) (*p < 0.05, Wilcoxon matched-pairs signed rank).

Mu, kappa and delta opioid receptors are expressed in the striatum where they regulate transmission through multiple circuits^27^,^28^,^29^. Although mu and delta opioid agonists induce reward behavior^30^, kappa opioid agonists are aversive in animal models^31^,^32^. Mu and delta receptors are both expressed postsynaptically on MSNs. Mu opioid receptors (MORs) are present on ChIs^29^,^33^,^34^,^35^ while kappa opioid receptors (KORs) are on dopamine terminals^36^,^37^. Both mu and kappa receptors have been shown to modulate striatal dopamine release^38^. To examine how these two opioid receptors regulate D2-IPSCs in MSNs, we applied agonists of either MORs or KORs while stimulating either dopamine terminals or ChIs. As MORs are located on CHIs, we found that bath application of the MOR antagonist DAMGO (1 μM) did not alter DAT-Cre:ChR2 evoked D2-IPSCs (2.3 ± 5% inhibition, n = 5, p > 0.05, W = 7, Wilcoxon) (Fig. 4A) but strongly decreased the amplitude of D2-IPSCs evoked by ChI stimulation (42.4 ± 5% inhibition, n = 6, W = 21 p < 0.05, Wilcoxon) (Fig. 4B). During these experiments, we also observed an outward current of 157 pA in one of six MSNs resulting from MOR activation on the MSN itself. The inhibition of ChAT-Cre:ChR2 evoked D2-IPSCs by DAMGO was reversed by the non-selective opioid receptor antagonist naloxone (1 μM, n = 6; p < 0.05 vs control, W = 17, Wilcoxon). This indicates that MORs on ChIs selectively modulate dopamine transmission release through the nicotinic pathway^38^. In contrast, the KOR agonist U69593 inhibited D2-receptor activation from both pathways (42.2 ± 4% inhibition of DAT-Cre:ChR2 evoked IPSCs, n = 6; 36.1 ± 6% inhibition of ChAT-Cre:ChR2 evoked IPSCs, n = 8; W = 21, p < 0.05 for both groups) (Fig. 5A and B). Thus, KORs equally modulate dopamine release when either dopamine terminals or ChIs are stimulated.

## Discussion

As dopamine release in the striatum is critical for motivated behaviors and action selection, modulation of this release is an important regulator of striatal circuitry. Independent of dopamine neuron impulse activity, nicotinic receptors on striatal dopamine terminals also facilitate dopamine release^5^,^11^. Using overexpressed GIRK2 channels in MSNs, we found that nicotinic receptors can modulate synaptic release of dopamine and subsequent D2-receptor activation. Direct comparison of D2-receptor activation in response to dopamine release driven through these two pathways revealed differential regulation of dopamine transmission.

Dopamine neurons typically fire at tonic low frequencies, and their firing switches to a bursting pattern in response to rewards and their cues^1^,^2^,^3^. Several studies have previously examined the role of nicotinic receptors as a high-pass filter of dopamine release at different firing frequencies using electrical stimulation^11^,^12^,^13^. This work has shown that nicotinic receptors strongly facilitate the release of dopamine at low stimulation frequencies (single pulse or 5 Hz) but have less of an effect^12^ or even depress dopamine release at higher frequencies (between 20 and 100 Hz)^13^. This nicotinic-mediated alteration of dopaminergic release probability was thought to result in contrast enhancement of dopamine release at different firing rates. However, dopamine neurons and ChIs show opposing firing patterns in response to salient stimuli^16^ and striatal dopamine release induces a pause in ChI tonic activity^22^,^23^. Thus, it is unlikely that dopamine neurons and ChIs would be simultaneously and synchronously active *in vivo*. We found that, similar to the electrochemical measurements of Zhang and Sulzer (2004), nicotinic receptors more strongly modulated dopamine release at low frequency electrical stimulation. However, by segregating these two pathways using optogenetics, we found that nicotinic receptors only act as high pass filters when both pathways are activated simultaneously. Selective stimulation of only dopamine terminals by activating ChR2 expressed in SNc axons of DAT-Cre mice revealed that D2-IPSCs exhibited no change in nicotinic receptor-mediated facilitation at any frequency examined. Thus, under conditions when dopamine neurons and ChIs are not synchronously active, nicotinic receptors failed to modulate the release of dopamine that underlies the activation of D2-receptors.

High-affinity α4β2 nicotinic receptors on midbrain dopamine neurons rapidly desensitize in response to low concentrations of ACh or nicotine^39^,^40^,^41^,^42^. As such, it is thought that these receptors on dopamine terminals readily desensitize in response to the concentrations of ACh that are released when multiple ChIs are simultaneously active^6^,^39^,^43^. The reduced contribution of this di-synaptic pathway during high frequency cholinergic stimulation enables nicotinic receptors to only respond to the first synchronous stimulus of a burst^5^,^11^. Thus, trains of optogenetic ChI stimuli failed to evoke further dopamine facilitation through nicotinic receptors when compared to single stimulation.

As DAT-Cre:ChR2 evoked D2-IPSCs facilitated during high frequency optogenetic stimulation and recovered from paired-pulse depression at a faster rate than ChAT-Cre:ChR2 evoked IPSCs, our results reveal a ChI-mediated mechanism of depression rather than dopamine vesicle depletion or D2-receptor desensitization. Similar to the observed recovery of ChAT-Cre:ChR2 evoked IPSCs, human α4β2 nAChRs expressed in heterologous systems can take ~20 seconds to recover their activity to normal levels in response to ACh and nicotine^26^. Further, ACh released onto MSNs at muscarinic synapses recovers within 10 seconds^18^, further suggesting that cholinergic vesicle depletion is unlikely to be responsible for the observed paired pulse depression of ChAT-Cre:ChR2 evoked IPSCs.

ChIs are tonically active both *in vivo* and in the absence of synaptic inputs^14^, largely due to the presence of hyperpolarization-activated cyclic nucleotide gated (HCN) channels and small-conductance potassium (SK) channels^44^,^45^. This has led to the idea that tonic ChI firing provides the striatum with a tone of ACh that activates postsynaptic receptors. We found that ACh tone in the slice provided little modulation of D2-receptor activation as DAT-Cre:ChR2 evoked IPSCs showed little change in amplitude in response to nicotinic receptor antagonists. Importantly, tonic ChI activity does not release sufficient amounts of ACh to desensitize nicotinic receptors, as optogenetic stimulation of ChIs could still evoke robust dopamine release. The lack of nicotinic modulation of DAT-Cre:ChR2 evoked IPSCs may result from super-physiological release of dopamine in response to light activated ChR2 conductance in the axon terminal. ChR2 is a light activated cation channel, stimulation of which causes an increased influx of Ca^2+^ in axon terminals compared with AP triggered release that has been shown to alter probability of release at other synapses^46^. As a result, one possibility may be that the effect of nicotinic receptors on dopamine terminals may have been occluded by ChR2-mediated release.

ACh tone may not affect dopamine release because acetylcholinesterase (AChE), the degradative enzyme of ACh, is enriched in the striatum and highly efficient, effectively terminating ACh signaling before it can activate striatal nicotinic receptors^6^. Thus, in agreement with previous electrochemical studies^11^, D2-IPSCs evoked from single action potentials in ChIs were unresolvable, whereas synchronous activity of ChIs is required to evoke D2-IPSCs. As AChE is integral for phasic ACh transmission at muscarinic synapses^18^, it likely degrades ACh rapidly such that tonic ChI firing is insufficient to drive dopamine release through nicotinic receptors. Thus, AChE may have a major role in titrating nicotinic receptor activity in the striatum, as its activity decreases striatal ACh while its inhibition desensitizes nicotinic receptors.

**Figure 6:**
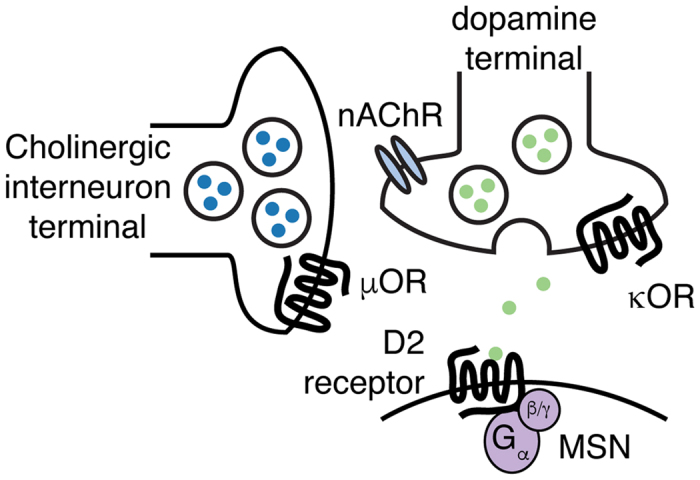
Microcircuit map of cholinergic and opioid modulation of dopamine release. Cholinergic terminals release ACh onto nicotinic receptors on dopamine terminals in the striatum. Synchronous ChI activity allows dopamine to be released through nicotinic-mediated facilitation. MORs present on ChI terminals modulate ChI-induced dopamine release, whereas KORs on dopaminergic terminals themselves regulate dopamine released through both pathways.

Within the striatum, mu, kappa and delta opioid receptors are widespread inhibitory modulators of striatal circuitry (Fig. 6). Enkephalin is released from indirect pathway MSNs and activates delta receptors largely on indirect pathway MSNs^4^,^27^. In addition, striatal enkephalin can activate striatal MORs on MSNs^27^ and ChIs^47^. Dynorphin released primarily from direct pathway MSNs activates KORs to drive either reward or aversion in different striatal compartments^48^. Electrochemical studies examining the effect of opioid receptors on striatal dopamine release have found that although KORs regulate dopamine release throughout the striatum, MOR regulation of dopamine release was spatially segregated to specific areas^38^. We similarly saw that KORs decreased dopamine release from both dopamine terminals and through ChI-mediated release. The areas of MOR regulation that others have observed could potentially be delineated by the striatal patch and matrix, as MORs are selectively expressed in the patch^19^,^49^ as is acetylcholinesterase^50^. ChIs are thought to reside either in the matrix or on the interface of striatal patch and matrix compartments^51^. However, they have far-reaching axons that cross the patch/matrix boundaries^52^. It is possible that we reliably observe MOR-mediated inhibition of ChAT-Cre:ChR2 evoked D2-IPSCs because convergent ChIs activated during optogenetic stimulation extend their axons from MOR-expressing patches.

Together, this work suggests that the role of the burst-firing pattern of ChIs common following reward stimuli is to synchronize firing, allowing for transient, phasic dopamine release, rather than to alter gain of dopamine release in the striatum. This parallels *in vivo* microdialysis studies that have shown that nicotinic receptor antagonism specifically in the striatum causes a small decrease in dopamine concentration over time^9^. Based on our results, this change in striatal dopamine levels may be differentially gated through opioid receptors. These results further provide insight into the regulation of dopamine release through two different mechanisms.

## Additional Information

**How to cite this article**: Mamaligas, A. A. *et al.* Nicotinic and opioid receptor regulation of striatal dopamine D2-receptor mediated transmission. *Sci. Rep.* **6**, 37834; doi: 10.1038/srep37834 (2016).

**Publisher's note:** Springer Nature remains neutral with regard to jurisdictional claims in published maps and institutional affiliations.
